# Dexamethasone to prevent postextubation airway obstruction in adults: a prospective, randomized, double-blind, placebo-controlled study

**DOI:** 10.1186/cc5957

**Published:** 2007-07-02

**Authors:** Chao-Hsien Lee, Ming-Jen Peng, Chien-Liang Wu

**Affiliations:** 1 Division of Pulmonary and Critical Care Medicine, Department of Internal Medicine, Mackay Memorial Hospital, No. 92, Section 2, Chung Shan North Road, Taipei City 104, Taiwan; 2 Mackay Medicine, Nursing and Management College, No.92, Shengjing Rd., Beitou District, Taipei City 112, Taiwan

## Abstract

**Introduction:**

Prophylactic steroid therapy to reduce the occurrence of postextubation laryngeal edema is controversial. Only a limited number of prospective trials involve adults in an intensive care unit. The purpose of this study was to ascertain whether administration of multiple doses of dexamethasone to critically ill, intubated patients reduces or prevents the occurrence of postextubation airway obstruction. Another specific objective of our study was to investigate whether an after-effect (that is, a transient lingering benefit) exists 24 hours after the discontinuation of dexamethasone.

**Methods:**

A randomized, placebo-controlled, double-blind trial was conducted in an adult medical intensive care unit of a tertiary care hospital. Eighty-six patients who had been intubated for more than 48 hours with a cuff leak volume (CLV) of less than 110 ml and who met weaning criteria were randomly assigned to receive either dexamethasone (5 mg; *n *= 43) or placebo (normal saline; *n *= 43) every six hours for a total of four doses on the day preceding extubation. CLV was measured before the first injection, one hour after each injection, and 24 hours after the fourth injection. Extubation was carried out 24 hours after the last injection. Postextubation obstruction (defined as the presence of stridor) was recorded within 48 hours of extubation.

**Results:**

Administration of dexamethasone during the 24-hour period preceding extubation resulted in a statistically significant increase in the CLV (*p *< 0.05). The significant increase of CLV and change of CLV relative to baseline tidal volume (percentage) occurred not only throughout the treatment period, but also 24 hours after the last dexamethasone injection. The incidence of postextubation stridor was significantly lower in the dexamethasone group than in the placebo group (10% [4/40] versus 27.5% [11/40]; *p *= 0.037), whereas there was no significant difference in reintubation rate between the two groups (2.5% [1/40] versus 5% [2/40]; *p *= 0.561).

**Conclusion:**

Prophylactic administration of multiple-dose dexamethasone is effective in reducing the incidence of postextubation stridor in adult patients at high risk for postextubation laryngeal edema. The after-effect of dexamethasone may validate the reduced incidence of postextubation stridor after multiple doses of dexamethasone.

**Trial registration:**

NCT00452062.

## Introduction

Laryngotracheal injury related to intubation may cause narrowing of the airway due to edema of the glottis. Laryngeal edema is more common after endotracheal intubation for more than 36 hours [1]. Edema in this region is associated with the increased risks for postextubation stridor, which increase reintubation rate. Reintubation may result in morbidity and mortality [2-6]. The prevalence of postextubation stridor ranges between 6% and 37% in intubated patients [7-12]. Because the presence of an endotracheal tube (ETT) precludes direct visualization of the upper airway, recognition of the edema due to laryngotracheal injury is often difficult. However, upper airway patency may be assessed indirectly in the intubated patient by cuff leak test. A reduced cuff leak volume (CLV) can predict the occurrence of laryngotracheal edema in high-risk patients [7-9,13]. Reports indicate that 1% to 17% of intensive care unit (ICU) patients develop postextubation airway obstruction requiring reintubation [3,4,9,11,14,15]. Mortality associated with reintubation has been estimated to be as high as 30% to 40% [4,6]. Factors correlating with the development of postextubation stridor include older age, female gender, elevated Acute Physiologic and Chronic Health Evaluation II score, low Glasgow Coma Scale score, excessive ETT size, and a prolonged intubation period [10,16-22].

Controversy still exists regarding the effectiveness of prophylactic steroid therapy for patients at risk for postextubation stridor [11,21-25]. Some studies involving postextubation stridor and analyses of outcomes for those receiving steroids during intubation have yielded inconclusive or negative results [11,23]. Only a limited number of randomized trials involving adults and evaluating the benefits of corticosteroid therapy prior to extubation have been conducted [21,22]. Moreover, studies regarding the efficacy of prophylactic corticosteroids for intubated patients have yielded conflicting results due to differences in the number of doses, types of corticosteroids, and timing and methods of administration to adult patients.

In our clinical practice, the extubation was usually performed one hour after the last injection of multiple prophylactic doses of dexamethasone in the patients with a CLV of less than 110 ml. Sometimes, due to unpredictable conditions, critically ill patients need to delay the planned extubation after steroid treatment. Previous studies reported that most high-risk patients susceptible to postextubation upper airway edema who fail extubation require reintubation within 48 to 72 hours [3,12]. However, little is known about the after-effect of multiple-dose dexamethasone to prevent postextubation stridor. The present study was conducted to evaluate the effects of prophylactic multiple-dose dexamethasone for adult ICU patients who had been intubated for more than 48 hours and who were undergoing their first elective extubation in an ICU setting. The specific objectives of our study were to determine whether multiple doses of dexamethasone are effective to reduce or prevent postextubation airway obstruction and to investigate whether an after-effect (that is, a transient lingering benefit) exists 24 hours after the discontinuation of dexamethasone.

## Materials and methods

### Patients

The current study included patients who were admitted to the adult medical ICU of the Mackay Memorial Medical Center (Taipei, Taiwan) between 1 October 2004 and 1 March 2006 and who met the inclusion criteria described below. Informed consent was obtained from the patients or their relatives prior to entrance in the trial. This study was approved by the Institutional Research Ethics Board.

All patients underwent endotracheal intubation with a high-volume, low-pressure cuffed tube possessing an internal diameter of 6.5, 7, 7.5, or 8 mm (Hi-Lo EVAC; Mallinckrodt Medical, Athlone, Ireland). Patients exhibiting excessive movement were sedated or paralyzed during mechanical ventilation. Routine nursing care included ETT suctioning every two hours and as needed to maintain a patent airway.

All patients were older than 18 years of age and met all of the following weaning criteria: (a) temperature of less than or equal to 38°C for more than eight hours, (b) discontinuous use of sedatives, (c) heart rate of more than or equal to 70 beats per minute and less than or equal to 130 beats per minute, (d) systolic blood pressure (SBP) of more than or equal to 80 mm Hg in the absence of vasopressor, (e) fraction of inspired oxygen (FiO_2_) of less than or equal to 60%, partial pressure of oxygen (PaO_2_) of more than or equal to 60 mm Hg, and PaO_2_/FiO_2 _ratio of more than 200, (f) positive end-expiratory pressure (PEEP) of less than or equal to 5 cm H_2_O, (g) rapid and shallow ratio of frequency to tidal volume of less than or equal to 105, (h) minute ventilation of less than or equal to 15 liters per minute, and (i) pH of more than or equal to 7.3. Supplemental oxygen was continued to maintain an oxygen saturation of more than 95% as measured by a pulse oximeter (Model 513; Novametrix Medical Systems Inc., Wallingford, CT, USA). The exclusion criterion was either (a) a history of extubation during the same hospitalization or (b) administration of corticosteroids seven days prior to extubation.

### Cuff leak test

The cuff leak test was therefore administered to the patients who required mechanical ventilation for more than 48 hours and who fit the above inclusion criteria. Patients were mechanically ventilated in the volume-assisted control mode by a Bird 8400 STi (Bird, a brand of VIASYS Healthcare Inc., Conshohocken, PA, USA) with a tidal volume of 10 ml/kg of ideal body weight, a respiratory rate of 20 breaths per minute, and a zero PEEP during CLV measurement. The operator-selected inspiratory tidal volume of 10 ml/kg of ideal body weight, displayed as an expiratory tidal volume, was recorded and the balloon cuff pressure was measured using a control inflator device (VBM Medizintechnik GmbH, Sulz am Neckar, Germany). The balloon cuff was deflated, the expiratory tidal volume was recorded over the six subsequent respiratory cycles, and the average of the lowest three values was used for subsequent analyses. The CLV was determined as the difference in the actual tidal volume before and after cuff deflation [7,10,13,21].

### Study protocol

Patients requiring mechanical ventilation for more than 48 hours and exhibiting a CLV of less than 110 ml before planned extubation were therefore included in the trial. Many investigators have recommended the use of a pre-extubation cuff leak test as a screening method for postextubation laryngeal edema [7,10,13,21], and selected studies have determined that a CLV of less than 110 ml is an available predictor of postextubation stridor. The cutoff point was based on a previous report by Miller and Cole [7]. A respiratory therapist, who was not involved in the patient care, used a table of a computer-generated randomization list with a block size of 4. This therapist also prepared the study drug and the identical-looking placebo and entered the data (including the cuff leak measurements) into a password-protected database.

Patients were randomly assigned to receive intravenous dexamethasone (Oradexon, N.V. Organon, Oss, The Netherlands) 5 mg per injection, or normal saline at an equivalent volume (placebo). The intubation was performed by the attending physicians and residents in the medical ward, emergency department, and medical ICU and prior to admission. Both the physician and the staff who administered the treatment (that is, dexamethasone or placebo) were blinded. The ICU physicians were not apprised of the measurements obtained by the respiratory therapist. On the day preceding extubation, dexamethasone or placebo was administered every six hours for a total of four doses. CLVs were measured before the first injection, one hour after each injection, and 24 hours after the fourth injection.

The patients in the non-intervention arm had extubation promptly after the cuff leak test. Extubation was performed 24 hours after the last injection of dexamethasone or placebo. Postextubation obstruction (defined as the presence of stridor heard with the aid of a stethoscope) was recorded within 48 hours of extubation. The presence of an audible, high-pitched wheeze was an indication for the inhalation of racemic epinephrine. The patients with respiratory distress were assigned to take non-invasive positive-pressure ventilation (bi-level positive airway pressure, Knightstar-335; Nellcor Puritan Bennett LLC, Plesanton, CA, USA) by face mask if they failed in response to two doses of epinephrine inhalation and exhibited at least two of the following criteria of respiratory distress: (a) respiratory acidosis (defined as an arterial pH of less than 7.35 with a partial pressure of arterial carbon dioxide of more than 45 mm Hg), (b) clinical signs suggestive of respiratory-muscle fatigue or increased respiratory effort (that is, use of accessory muscles, intercostal retraction, or paradoxical motion of the abdomen), (c) a respiratory rate of more than 25 breaths per minute for two consecutive hours, and (d) hypoxemia (defined as an arterial oxygen saturation of less than 90% or a PaO_2 _of less than 80 mm Hg with an FiO_2 _of more than 50%).

Patients were reintubated with mechanical ventilation support if they met at least one of the following criteria: (a) a pH of less than 7.3 with a partial pressure of carbon dioxide increase of more than 15 mm Hg, (b) a change in mental status rendering the patient unable to tolerate non-invasive ventilation, (c) a decrease in the oxygen saturation to less than 85% despite the use of a high FiO_2 _(a PaO_2 _of less than 50 mm Hg with an FiO_2 _of more than 70%), (d) lack of improvement in signs of respiratory-muscle fatigue, (e) hypotension with an SBP of less than 80 mm Hg for more than 30 minutes despite adequate volume challenge, (f) a diastolic blood pressure drop of more than 20 mm Hg, or (g) copious secretions that could not be cleared adequately or that were associated with acidosis, hypoxemia, or changes in mental status (somnolence, agitation, or diaphoresis).

### Statistical analysis

The sample size calculation was based on the preliminary data obtained in the medical ICU of our hospital. During a six month period, 45 patients with a CLV of less than 110 ml underwent planned extubation and 25 of the patients received dexamethasone every six hours for a total of four doses in 24 hours, as decided by the attending physician. Extubation was performed 24 hours after the last dose of dexamethasone. Postextubation stridor occurred in 1 of the 25 patients given corticosteroids (4%) and in 7 of 20 patients not given the drug (35%). A sample size of 60 subjects was therefore calculated for a two-sided test with a type 1 error of 5% and a power of 90%.

Statistical analyses were conducted using the SPSS software package (version 13.0; SPSS Inc., Chicago, IL, USA). Univariate analyses between the dexamethasone and placebo groups were conducted using Student *t *tests for continuous variables and a Pearson chi-square test for categorical variables, or the Fisher exact test, as indicated. For all tests, a *p *value of less than 0.05 was considered statistically significant.

## Results

Of all the 386 patients going through the CLV test, 300 had a CLV of more than or equal to 110 ml and comprised the non-intervention arm. Fifteen patients were excluded because of self-extubation (*n *= 6) or a deterioration in clinical status (*n *= 9). The patients in the non-intervention group were extubated at once after the cuff leak test.

**Figure 1:**
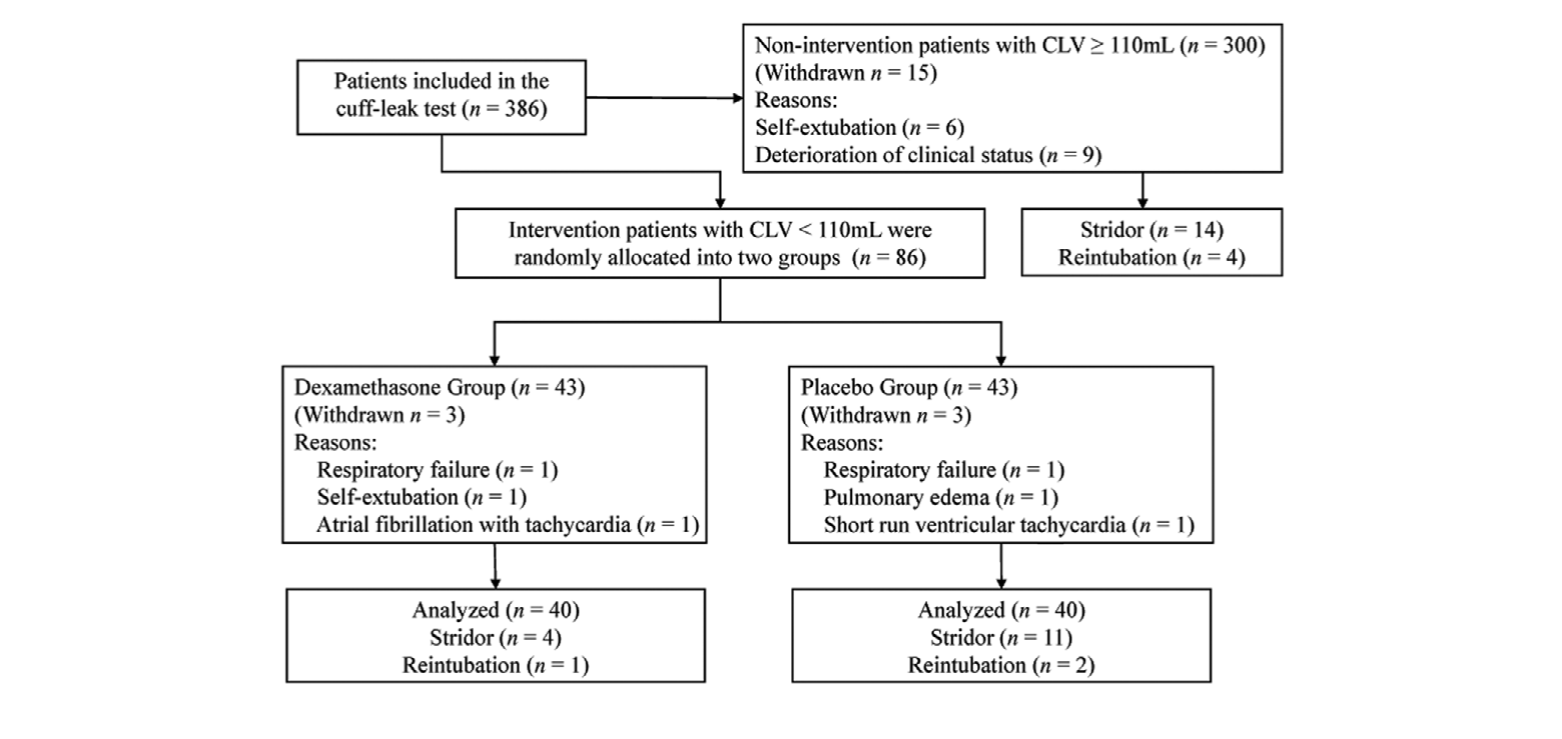
Study flowchart. CLV, cuff leak volume.

Eighty-six patients with a CLV of less than 110 ml met the inclusion criteria. These patients comprised the intervention arm and were randomly assigned into the dexamethasone group (*n *= 43) and the placebo group (*n *= 43). In the dexamethasone group, 3 patients withdrew due to respiratory failure (*n *= 1), self-extubation (*n *= 1), and atrial fibrillation with tachycardia (*n *= 1). In the placebo group, 3 patients withdrew due to respiratory failure (*n *= 1), pulmonary edema (*n *= 1), and a short run of ventricular tachycardia (*n *= 1). A total of 80 patients were therefore analyzed. The randomized disposition of the patients is shown in Figure 1. The dexamethasone and placebo groups did not significantly differ in the demographic characteristics, including age, gender, weight, height, duration of intubation, diameter of ETT, Glasgow Coma Scale score, underlying disease, and severity of illness (Table 1).

**Table 1:** Demographic data of patients.

	Dexamethasone group (*n *= 40)	Placebo group (*n *= 40)	*P *value
Age (years)^a^	72.4 ± 14.7	72.7 ± 13.8	0.55
Gender^b^			0.66
Female	34 (85)	32 (88)	
Male	6 (15)	8 (12)	
Body weight (kg)^a^	58.6 ± 9.6	57.5 ± 10.6	0.68
Height (cm)^a^	155.8 ± 6.9	152.9 ± 7.3	0.10
APACHE II score^a^	19.5 ± 2.9	21.5 ± 3.7	0.08
Albumin (g/dl)^a^	2.6 ± 0.6	2.5 ± 0.6	0.98
Hemoglobin (g/dl)^a^	9.7 ± 1.7	9.4 ± 1.3	0.30
Internal diameter of endotracheal tube^b^			0.14
6.5 or 7 mm	5 (12)	4 (10)	
7.5 or 8.0 mm	35 (88)	36 (90)	
Intubation time (hours)^a^	167.3 ± 48.1	158.5 ± 49.0	0.95
Reason for intubation^b^			0.94
Pneumonia	11 (28)	9 (23)	
Sepsis	4 (10)	5 (13)	
Heart failure	4 (10)	5 (13)	
Acute respiratory distress syndrome	2 (5)	3 (8)	
Chronic obstructive pulmonary disease	5 (13)	4 (10)	
Asthma	0	1 (3)	
Other	14 (35)	13 (33)	
Glasgow Coma Scale score			0.65
3–8	11	10	
9–12	14	11	
13–15	15	19	

^a^Data are presented as mean ± standard deviation; ^b^data are presented as number of patients (percentage of patients). APACHE II, Acute Physiologic and Chronic Health Evaluation II.

**Figure 2:**
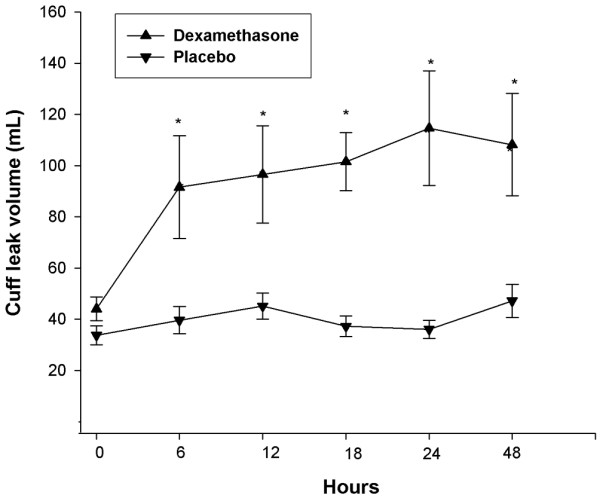
Cuff leak volumes for critically ill patients receiving injections of dexamethasone or placebo (normal saline) before extubation. Patients who had been intubated for more than two days and who exhibited cuff leak volumes of less than 110 ml at T = 0 received injections at 6, 12, 18, and 24 hours, followed by extubation. Cuff leak tests were measured at T = 0, 1 hour after each injection, and 24 hours after the fourth injection. Differences in cuff leak volumes between the two groups were significant (*P < 0.05).

**Figure 3:**
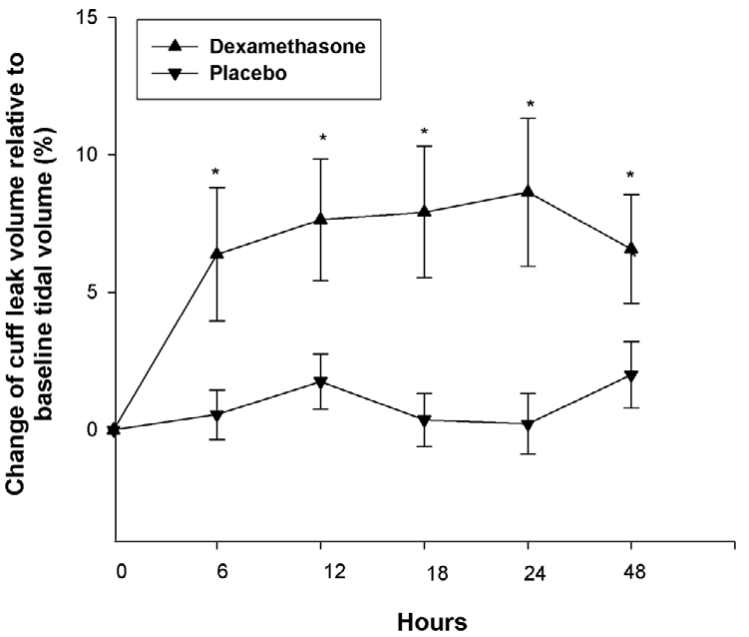
Cuff leak volumes for critically ill patients receiving injections of dexamethasone or placebo (normal saline) before extubation. Patients who had been intubated for more than two days and who exhibited cuff leak volumes of less than 110 ml at T = 0 received injections at 6, 12, 18, and 24 hours, followed by extubation. Differences in change of cuff leak volume relative to baseline tidal volume (percentage) between the two groups were significant (*P < 0.05).

The measurements of CLV after each injection of dexamethasone or placebo, given at the interval of six hours, are shown in Figure 2, and change of CLV relative to baseline tidal volume (percentage) is shown in Figure 3. Compared to the placebo group, a significant increase of CLV was noted in the dexamethasone group. The significant difference initially manifested at the first dexamethasone injection and was consistently noted after each dexamethasone injection. At the end of our observation period (that is, 24 hours after the last injection), the CLV in the dexamethasone group was still significantly higher than in the placebo group. Thus, the effect of multiple-dose dexamethasone in increasing CLV occurred not only throughout the treatment period, but also in the observation period of 24 hours after the last dexamethasone injection.

The incidence of postextubation stridor was 4.9% (14/285) in the non-intervention arm, 10% (4/40) in the dexamethasone group, and 27.5% (11/40) in the placebo group. The absolute risk reduction and number needed to treat for postextubation stridor were 18% and 5.7, respectively. Postextubation stridor was significantly different between the dexamethasone and the placebo groups (*p *= 0.037; odds ratio [OR] for stridor: 0.214; 95% confidence interval [CI]: 0.055 to 0.838). Each of the 29 patients with stridor received epinephrine by inhalation, and 24 of these patients underwent non-invasive positive-pressure ventilation: 3 in the dexamethasone group (7.5%), 9 in the placebo group (22.5%), and 12 in the non-intervention arm (4.2%).

Reintubation with mechanical support was necessary in 2.5% (1/40) of patients in the dexamethasone group, 5% (2/40) in the placebo group, and 1.4% (4/285) in the non-intervention arm. However, the difference in the reintubation rate was not statistically significant between the dexamethasone and the placebo groups (*p *= 0.56; OR for reintubation: 0.49; 95% CI: 0.04 to 5.60). During the period of our study, no patients exhibited gastrointestinal bleeding, gastric pain or irritation, acne, psychiatric disturbances (including personality changes, irritability, euphoria, and mania), or cushingoid features.

## Discussion

The potential benefit of steroids to laryngeal edema is presumably based on its anti-inflammatory actions, which inhibit the release of inflammatory mediators and decrease capillary permeability. The risk of harm from steroid therapy for 24 hours or less to prevent postextubation laryngeal edema is negligible [22,25,26]. The extent of the effect of prophylactic steroids on airway obstruction is still a matter of some controversy. A single injection of dexamethasone (1 mg/kg) one hour before extubation had no effect on subglottic injury in a rabbit model [27]. In a study on unselected adult patients, an 8-mg injection of dexamethasone given one hour before extubation did not reduce the number of patients requiring reintubation [1]. Another study on children reported that six doses of dexamethasone (0.5 mg/kg) given every six hours starting 6 to 12 hours before extubation had no effect on the incidence of postextubation stridor [23]. Contrary to the previous studies, the positive results were reported by other studies, most of which were conducted on pediatric patient populations. Four doses of dexamethasone (0.25 mg/kg) given at eight hour intervals could prevent stridor in preterm infants [28]. Six doses of dexamethasone (0.5 mg/kg) given every six hours were also effective in preventing postextubation stridor for intubated children in the ICU [24]. A meta-analysis concluded that prophylactic dexamethasone reduces postextubation stridor in infants and children [25]. In a comparison study, one 2-mg dose of nebulized budesonide was as effective as a single intravenous dose of dexamethasone (0.6 mg/kg) in treating symptoms in children with croup [29].

We conducted this randomized clinical trial to evaluate the effects of prophylactic dexamethasone therapy in preventing laryngeal edema for adult patients with a CLV of less than 110 ml in an adult medical ICU setting. The choice of dexamethasone was based on its high anti-inflammatory potency, negligible mineralocorticoid effects at therapeutic doses, and long duration of action [30]. To our knowledge, this was the first study to periodically monitor the CLV after each dexamethasone administration and to investigate the after-effect until 24 hours after the last dose of dexamethasone. Our results revealed the significant reduction of postextubation stridor (10% in the dexamethasone group and 27.5% in the placebo group) and reintubation rate (2.5% in the dexamethasone group and 5% in the placebo group). Moreover, CLV and the ratio of CLV to tidal volume significantly increased, and this effect occurred not only throughout the treatment period, but also 24 hours after the last dexamethasone injection.

Our findings of steroid effect in reducing postextubation stridor were consistent with the results of the two recent studies in adult medical and surgical ICU settings [21,22]. Cheng and colleagues [21] reported that low CLV with less than 24% of tidal volume could be a useful predictor of postextubation stridor. Four prophylactic infusions of 40 mg of methylprednisolone, given at six hour intervals to high-risk adult patients with low CLV, significantly decreased the incidence of postextubation stridor from 30.2% to 7.1% and the reintubation rate from 18.6% to 7.1% [21]. In a recent study with a large number of subjects, Francois and colleagues [22] reported that four doses of 20 mg of methylprednisolone, given at four hour intervals, significantly reduced the incidence of postextubation stridor from 22% to 3% and reduced the incidence of reintubation from 8% to 4%. However, the subjects in this study were not restricted to the high-risk patients for postextubation laryngeal edema (unlike the subjects in our study).

The effects in the reduction of postextubation stridor may be influenced by varied cutoff points based on CLV. Cheng and colleagues [21] used low CLV with less than 24% of tidal volume and set the tidal volume at 8 mg/kg of ideal body weight. However, we adopted the criterion of CLV of less than 110 ml and the tidal volume was set at 10 ml/kg of ideal body weight [7,10,13]. The reduction of postextubation stridor in our study was statistically significant, but not as dramatic as that of the study of Cheng and colleagues. Another reason for this discrepancy may be the difference in the timing of extubation. Cheng and colleagues executed the extubation one hour after methylprednisolone administration over the span of 24 hours. To monitor the CLV level and check the after-effect of dexamethasone administration for 24 hours, we delayed the extubation until 24 hours after the last dexamethasone injection. The after-effect of dexamethasone validates the reduced incidence of postextubation stridor after multiple doses of dexamethasone.

In regard to the reintubation rate, there was no significant difference between the dexamethasone-treated and the placebo groups in our study, whereas the reintubation rates of our subjects (2.5% in the dexamethasone group and 5% in the placebo group) were lower than those of Cheng and colleagues [21] (7.1% in the methylprednisolone group and 18.6% in the placebo group) and Francois and colleagues [22] (4% in the methylprednisolone group and 8% in the placebo group). The different results among these studies may be explained by the difference in the types of corticosteroids, risk factors predisposing to laryngeal edema, monitoring duration after extubation, and treatments for postextubation stridor.

Our study and the other two studies mentioned here substantiated that corticosteroids confer the benefits on adult patients at high risk for postextubation laryngeal edema, whereas a routine prophylactic use of corticosteroids to prevent postextubation stridor in every intubated patient is unwarranted. Dexamethasone and other steroids, in appropriate doses, can be helpful in alleviating laryngeal edema in intubated high-risk patients susceptible to airway obstruction, such as those requiring repeated or prolonged intubations.

## Conclusion

Dexamethasone, a long-acting and potent corticosteroid, is suitable for preventing postextubation airway edema. Administration of multiple prophylactic doses of dexamethasone significantly decreases the incidence of postextubation stridor in adult patients at high risk to develop airway obstruction. The after-effect of dexamethasone may validate the reduced incidence of postextubation stridor after multiple-dose dexamethasone.

## Key messages

• Cuff leak volumes for patients at high risk for postextubation upper airway edema are increased by administration of dexamethasone during the 24 hours preceding extubation.

• The incidence of postextubation stridor in these dexamethasone-treated patients is significantly reduced.

## Abbreviations

CI = confidence interval; CLV = cuff leak volume; ETT = endotracheal tube; FiO_2 _= fraction of inspired oxygen; ICU = intensive care unit; OR = odds ratio; PaO_2 _= partial pressure of oxygen; PEEP = positive end-expiratory pressure; SBP = systolic blood pressure.

## Competing interests

The authors declare that they have no competing interests.

## Authors' contributions

C-HL designed and supervised the research; collected, analyzed, and interpreted the data; and drafted and revised the manuscript. M-JP contributed to the conception of the study and coordinated the project. C-LW made substantial contributions to the conception and design of the study and approved the final version of the manuscript.

## References

[B1] Darmon JY, Rauss A, Dreyfuss D, Bleichner G, Elkharrat D, Schlemmer B, Tenaillon A, Brun-Buisson C, Huet Y (1992). Evaluation of risk factors for laryngeal edema after tracheal extubation in adults and its prevention by dexamethasone. A placebo-controlled, double-blind, multicenter study. Anesthesiology.

[B2] Demling RH, Read T, Lind LJ, Flanagan HL (1988). Incidence and morbidity of extubation failure in surgical intensive care patients. Crit Care Med.

[B3] Esteban A, Alia I, Tobin MJ, Gil A, Gordo F, Vallverdu I, Blanch L, Bonet A, Vazquez A, de Pablo R (1999). Effect of spontaneous breathing trial duration on outcome of attempts to discontinue mechanical ventilation. Spanish Lung Failure Collaborative Group. Am J Respir Crit Care Med.

[B4] Epstein SK, Ciubotaru RL, Wong JB (1997). Effect of failed extubation on the outcome of mechanical ventilation. Chest.

[B5] Torres A, Gatell JM, Aznar E, el-Ebiary M, Puig de la Bellacasa J, Gonzalez J, Ferrer M, Rodriguez-Roisin R (1995). Reintubation increases the risk of nosocomial pneumonia in patients needing mechanical ventilation. Am J Respir Crit Care Med.

[B6] Esteban A, Alia I, Gordo F, Fernandez R, Solsona JF, Vallverdu I, Macias S, Allegue JM, Blanco J, Carriedo D (1997). Extubation outcome after spontaneous breathing trials with T-tube or pressure support ventilation. The Spanish Lung Failure Collaborative Group. Am J Respir Crit Care Med.

[B7] Miller RL, Cole RP (1996). Association between reduced cuff leak volume and postextubation stridor. Chest.

[B8] Sandhu RS, Pasquale MD, Miller K, Wasser TE (2000). Measurement of endotracheal tube cuff leak to predict postextubation stridor and need for reintubation. J Am Coll Surg.

[B9] Kemper KJ, Benson MS, Bishop MJ (1991). Predictors of postextubation stridor in pediatric trauma patients. Crit Care Med.

[B10] Jaber S, Chanques G, Matecki S, Ramonatxo M, Vergne C, Souche B, Perrigualt PF, Eledjam JJ (2003). Postextubation stridor in intensive care unit patients. Risk factors evaluation and importance of the cuff-leak test. Intensive Care Med.

[B11] Ho LI, Harn HJ, Lien TC, Hu PY, Wang JH (1996). Postextubation laryngeal edema in adults. Risk factor evaluation and prevention by hydrocortisone. Intensive Care Med.

[B12] Bladimir G, Fernando FV, Andres E (2003). Deleterious effects of reintubation of mechanically ventilated patients. Clin Pulmon Med.

[B13] Chung YH, Chao TY, Chiu CT, Lin MC (2006). The cuff-leak test is a simple tool to verify severe laryngeal edema in patients undergoing long-term mechanical ventilation. Crit Care Med.

[B14] Efferen LS, Elsakr A (1998). Postextubation stridor: risk factors and outcome. J Assoc Acad Minor Phys.

[B15] Vallverdu I, Calaf N, Subirana M, Net A, Benito S, Mancebo J (1998). Clinical characteristics, respiratory functional parameters, and outcome of a two-hour T-piece trial in patients weaning from mechanical ventilation. Am J Respir Crit Care Med.

[B16] Koka BV, Jeon IS, Andre JM, MacKay I, Smith RM (1977). Postintubation croup in children. Anesth Analg.

[B17] Bishop MJ (1989). Mechanisms of laryngotracheal injury following prolonged tracheal intubation. Chest.

[B18] Ferdinande P, Kim DO (1995). Prevention of postintubation laryngotracheal stenosis. Acta Otorhinolaryngol Belg.

[B19] Erginel S, Ucgun I, Yildirim H, Metintas M, Parspour S (2005). High body mass index and long duration of intubation increase postextubation stridor in patients with mechanical ventilation. Tohoku J Exp Med.

[B20] Kastanos N, Estopa Miro R, Marin Parez A, Xaubet Mir A, Agusti-Vidal A (1983). Laryngotracheal injury due to endotracheal intubation: incidence, evolution, and predisposing factors. A prospective long-term study. Crit Care Med.

[B21] Cheng KC, Hou CC, Huang HC, Lin SC, Zhang H (2006). Intravenous injection of methylprednisolone reduces the incidence of postextubation stridor in intensive care unit patients. Crit Care Med.

[B22] Francois B, Bellisant E, Gissot V, Desachy S, Boulain T, Preux P-M, Vignon P (2007). 12-h pretreatment with methylprednisolone versus placebo for prevention of postextubation laryngeal oedema: a randomised double-blind trial. Lancet.

[B23] Tellez DW, Galvis AG, Storgion SA, Amer HN, Hoseyni M, Deakers TW (1991). Dexamethasone in the prevention of postextubation stridor in children. J Pediatr.

[B24] Anene O, Meert KL, Uy H, Simpson P, Sarnaik AP (1996). Dexamethasone for the prevention of postextubation airway obstruction: a prospective, randomized, double-blind, placebo-controlled trial. Crit Care Med.

[B25] Markovitz BP, Randolph AG (2002). Corticosteroids for the prevention of reintubation and postextubation stridor in pediatric patients: a meta-analysis. Pediatr Crit Care Med.

[B26] Hawkins DB, Crockett DM, Shum TK (1983). Corticosteroids in airway management. Otolaryngol Head Neck Surg.

[B27] Kil HK, Alberts MK, Liggitt HD, Bishop MJ (1997). Dexamethasone treatment does not ameliorate subglottic ischemic injury in rabbits. Chest.

[B28] Couser RJ, Ferrara TB, Falde B, Johnson K, Schilling CG, Hoeckstra RE (1992). Effectiveness of dexamethasone in preventing extubation failure in preterm infants at increased risk for airway edema. J Pediatr.

[B29] Geelhoed GC, Macdonald WB (1995). Oral and inhaled steroids in croup: a randomized, placebo-controlled trial. Pediatr Pulmonol.

[B30] Schimmer BP, Parker KL, Hardman JG, Gilman AG, Limbird LE (1996). Adrenocorticotropic hormones, adrenocortical steroids and their synthetic analogues; inhibitors of the synthesis and actions of adrenocortical hormones. Goodman and Gilman's the Pharmacological Basis of Therapeutics.

